# Prognostic Value of Positron Emission Tomography in Advanced Cholangiocarcinoma: A Single-Institution Study and Literature Review

**DOI:** 10.7759/cureus.31026

**Published:** 2022-11-02

**Authors:** Justin L Pevner, Tawee Tanvetyanon

**Affiliations:** 1 Department of Clinical Oncology, USF (University of South Florida) Health Morsani College of Medicine, Tampa, USA; 2 Department of Thoracic Oncology, H. Lee Moffitt Cancer Center, Tampa, USA

**Keywords:** cancer, cholangiocarcinoma, prognosis, survival, positron emission tomography

## Abstract

Background: Cholangiocarcinoma is an uncommon malignancy in Western countries. Previous studies from mostly Asian countries have suggested the prognostic value of 18F-fluorodeoxyglucose positron-emission tomography integrated with computerized tomography (PET/CT) for advanced cholangiocarcinoma. Here, we investigated the prognostic value of PET/CT at our institution.

Methods: A retrospective cohort study based on medical record review was conducted for patients with advanced cholangiocarcinoma who underwent treatment from January 2009 to January 2019 at a large academic institution in the United States. The outcomes of interest were overall survival. Multivariable analyses were performed to characterize the relationship between survival and the highest maximum standardized uptake value (SUVmax) from baseline PET/CT scans.

Results: Analyses included data from 61 patients. The median age was 68.9 years and 87% of patients were White. The median highest SUVmax was 8.7 (range: background value to 22.8). In a multivariable analytic model including SUVmax, patient demography, and baseline laboratory parameters, SUVmax was identified as one of the independent predictors of survival higher SUVmax significantly predicted worse survival, with hazard ratio 2.26 (95% CI: 1.07-4.75, *p*=0.03). White race, higher albumin level, younger age, and lower cancer antigen (CA) 19-9 levels were associated with a decreasing risk of death.

Conclusion: In this single-institution retrospective analysis, we found that baseline SUVmax was one of the significant prognostic factors for patients with advanced cholangiocarcinoma.

## Introduction

Cholangiocarcinoma is an uncommon malignancy in Western countries. According to the data from the Surveillance Epidemiology and End Results (SEER) program, the incidence of cholangiocarcinoma is estimated to be 1.26 per 100,000 people per year in the United States [1]. This number is in sharp contrast with certain Asian countries such as Thailand and China, where the incidence can approach 14.0 per 100,000 people per year [2]. Most patients diagnosed with cholangiocarcinoma have a poor prognosis. While surgery can be curative for some, over two-thirds of patients will not be a surgical candidate due to distant metastasis, extension into the peritoneal cavity, or lack of an adequate liver remnant [3,4]. For patients with advanced cholangiocarcinoma, the mainstay of treatment is systemic chemotherapy, which is only modestly effective. The standard front-line gemcitabine-based chemotherapy regimen typically yields a median survival of approximately 11-12 months [5].

Several studies have identified factors that can be useful in providing prognostic information for patients with advanced cholangiocarcinoma. These include tumor marker level [6], liver function, presence of liver metastasis [7], and performance status [8]. In recent years, the use of 18F-fluorodeoxyglucose (FDG) positron-emission tomography integrated with computerized tomography (PET/CT) has been found useful for the staging of patients with early-stage cholangiocarcinoma [9,10]. Furthermore, among patients who have had surgical resection, the baseline metabolic parameters from PET/CT obtained prior to surgery can be prognostic [11,12]. Nevertheless, to date, the clinical role of PET/CT in the care of patients with advanced, unresectable cholangiocarcinoma remains unclear. Some studies have suggested that the degree of hypermetabolism or reduction of hypermetabolism after chemotherapy can provide prognostic information [13,14]. For example, a maximum standard uptake value (SUVmax) of ≥5 has been associated with poor survival [13]. However, interpretation of the results can be challenging due to the inclusion of patients with early-stage disease or gallbladder carcinoma in the analysis [15,16]. Furthermore, it should also be noted that the available studies were conducted mostly in Asian countries. Given the potential difference in drug metabolism and healthcare system, the findings from Asian countries may not necessarily be generalizable to a population in Western countries.

Over the years, our institution has performed PET/CT for patients with advanced cholangiocarcinoma prior to systemic chemotherapy. In this study, we retrospectively investigated the prognostic value of PET/CT for the survival of patients with advanced cholangiocarcinoma.

## Materials and methods

Patient cohort

After receiving an exempted approval from the scientific review committee and institutional review board of the University of South Florida (STUDY000130, protocol MCC20396), the institutional tumor registry was searched for patients with a diagnosis of advanced cholangiocarcinoma treated at Moffitt Cancer Center between January 1, 2009, and January 1, 2019. Eligible patients were those with advanced disease, defined as those with distant metastatic disease or having recurrent and/or persistent disease following surgery or definitive chemoradiation. Patients with at least one available PET/CT scan performed before commencing on palliative systemic therapy or embolization therapy were eligible to be included for analyses. Patients with hepatocellular or gallbladder carcinoma, those who had no available PET/CT, or those without semiquantitative measurement reflecting SUVmax were excluded from analyses.

Definition and data abstraction

The earliest available PET/CT report for each patient was reviewed for reported hypermetabolic lesions in the liver, extrahepatic bile ducts, lymph nodes, or extra-hepatobiliary metastatic sites. The highest reported SUVmax value was recorded. During the study period, PET/CT at our institution was performed using a Siemens Biograph PET/CT scanner with CT images for localization (Model CTI 3201958-00; Siemens Medical Solution, Malvern, PA). Patients underwent an overnight fast and glucose testing to ensure the level was below 175 mg/dL. Patients then received an 18-FDG injection, with the scan starting approximately 90 minutes after the injection. Fusion images were evaluated using a semiquantitative method via SUV, which reflects the ratio of concentrated activity to whole-body average radioactivity. SUVmax was measured using a volumetric region-of-interest technique with an image analysis software (SynGo VX49b-Leonardo VD30B; Siemens AG, Berlin, Germany). The highest SUVmax among these sites was also recorded. Additional patient information including comorbidities and treatment information was obtained. The albumin, cancer antigen 19-9 (CA 19-9), and total bilirubin values available closest to the date of the PET/CT were recorded. The comorbidity index was calculated according to Charlson’s index.

Statistical consideration

Descriptive statistics including median and range for scale variables as well as count and percentage for categorical variables were used. The nonparametric Mann-Whitney test was used to compare continuous variables while Pearson’s Chi-square or Fisher’s Exact tests were used to compare categorical variables as appropriate. The outcomes of interest were overall survival from PET/CT, calculated from the date of the PET/CT to the date of death or last known alive, as well as overall survival from diagnosis, calculated from the date of initial pathological diagnosis. Death was verified from medical records or the social security death index. Survival was estimated using the method of Kaplan-Meier. Log-rank test was used to compare the median survival between groups. Multi-variable Cox proportional hazard models were fitted to evaluate the impact of clinical factors on survival. The backward stepwise selection method was used to identify independent predictors of survival. All statistical analyses and graphic illustrations were performed using IBM SPSS Statistics for Windows, Version 26.0 (Released 2019; IBM Corp., Armonk, New York, United States).

## Results

Patient characteristics

An initial search of the institutional tumor registry yielded 149 patients with advanced cholangiocarcinoma during the study period. Following the study inclusion and exclusion criteria, data from 61 patients were found eligible for analyses. One patient had a pathological diagnosis of combined hepatocellular-cholangiocarcinoma. The patient characteristics are summarized below (Table 1).

**Table 1:** Patient characteristics * Data available from 33 patients with primary tumor measured ^†^Two patients received unidentified regimen(s) of systemic chemotherapy at outside institutions SUVmax, maximum standard uptake value; CA 19-9. cancer antigen 19-9; FDG, 18-fluorodeoxyglucose

Clinical characteristics	Total patients N=61 (%)	Lower SUVmax N=30 (%)	Higher SUVmax N=31 (%)	p-value
Median age years, range	68.9, 35.1-86.8	66.1, 44.4-86.8	71.5, 35.0-81.6	0.12
Location:				0.15
-Extrahepatic	21 (34)	13 (43)	8 (26)	
-Intrahepatic	40 (66)	17 (57)	23 (74)	
Distant metastasis at baseline:				0.92
-Absent	39 (64)	19 (63)	20 (65)	
-Present	22 (36)	11 (37)	11 (35)	
Median primary tumor size cm*, range	7.4, 1.0-13.9	5.4, 1.9-11.0	8.3, 1.0-13.9	0.14
Median total bilirubin mg/dL, range	0.6, 0.2-22.5	0.8, 0.3-22.5	0.5, 0.2-8.0	0.04
Median CA 19-9 U/mL, range	207.4, 0.6-56838.0	340.0, 0.7-46705.0	93.5, 0.6-56838.0	0.04
Median albumin g/dL, range	4.0, 2.6-4.7	3.9, 2.6-4.7	4.1, 3.1-4.7	0.28
Comorbidity index:				0.45
-0	33 (54)	18 (60)	15 (48)	
-1	21 (34)	8 (27)	13 (42)	
-≥2	7 (12)	4 (13)	3 (10)	
Sex:				0.69
-Female	28 (46)	13 (43)	15 (48)	
-Male	33 (54)	17 (57)	16 (52)	
Race:				0.47
-White race	53 (87)	25 (83)	28 (90)	
-Other races	8 (13)	5 (17)	3 (10)	
PET/CT before chemotherapy:				0.21
-Yes	54 (89)	25 (83)	29 (93)	
-No	7 (11)	5 (17)	2 (7)	
Systemic chemotherapy:†				0.81
-None	9 (15)	4 (14)	5 (17)	
-1 line	26 (44)	12 (41)	14 (47)	
-≥2 lines	24 (41)	13 (45)	11 (37)	
Radioembolization:				0.47
-No	52 (85)	27 (90)	25 (81)	
-Yes	9 (15)	3 (10)	6 (19)	
Chemoradiation:				0.03
-No	53 (87)	23 (77)	30 (97)	
-Yes	8 (13)	7 (23)	1 (3)	
Prior liver or surgical resection:				1.00
-No	55 (90)	27 (90)	28 (90)	
-Yes	6 (10)	3 (10)	3 (10)	
Median FDG dosage mSV, range	11.0, 8.1-19.5	10.8, 8.1-19.5	11.1, 8.3-16.9	0.61

The vast majority of patients identified as White (87%) and received systemic chemotherapy (85%). PET/CT scans were acquired before initiation of systemic therapy except for seven patients (11%), who had a history of prior systemic therapy with or without radiotherapy. The most common first-line systemic therapy was gemcitabine with or without cisplatin, followed by gemcitabine plus oxaliplatin. The most common second-line systemic therapy was 5-fluorouracil plus an oxaliplatin-based regimen or 5-fluorouracil with or without radiotherapy. Treatment with immune checkpoint inhibitor occurred in seven patients.

SUVmax

Among 61 patients analyzed, SUVmax was reported from the primary tumor in 56 patients (92%), from a regional node in 33 patients (54%), and from a distant metastatic lesion in 22 patients (36%). The median SUVmax values in these lesions were 8.7, 5.9, and 5.1, respectively. For each patient, the lesion with the highest SUVmax was then included in the analysis. The median highest SUVmax was 8.7 (range: background uptake value to 22.8).

For analytic purposes, the patients were divided into two groups based on median SUVmax, with lower as <8.7 and higher as ≥8.7. There were 30 and 31 patients in each group, respectively. The characteristics of patients were comparable between the two groups, except for total bilirubin level and CA 19-9 level in that there was a significant tendency for higher total bilirubin and CA 19-9 in the lower SUVmax group.

Survival outcomes

At the time of analysis, 60 patients had died. The median survival as calculated from the date of the PET/CT scan was 11.5 months (95% CI 9.9-13.1). The median survival as calculated from the date of diagnosis was 11.9 months (95% CI 9.6-14.2).

**Figure 1:**
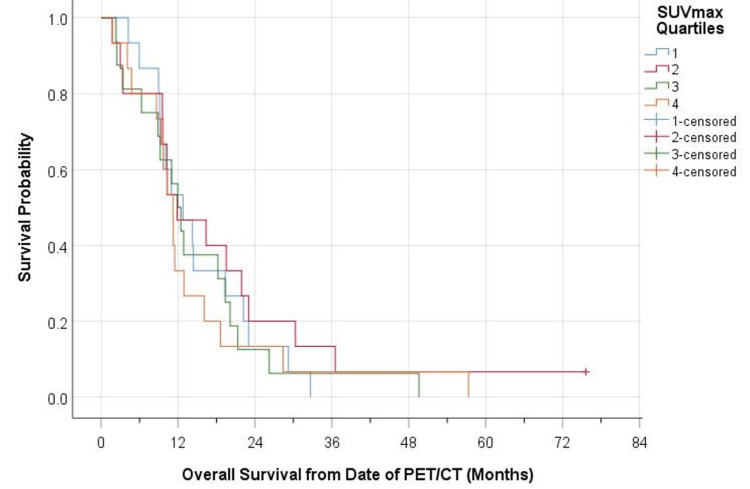
Relationship between SUVmax and overall survival from the date of PET/CT SUVmax, maximum standard uptake value; PET/CT, positron emission tomography/ computerized tomography

**Figure 2:**
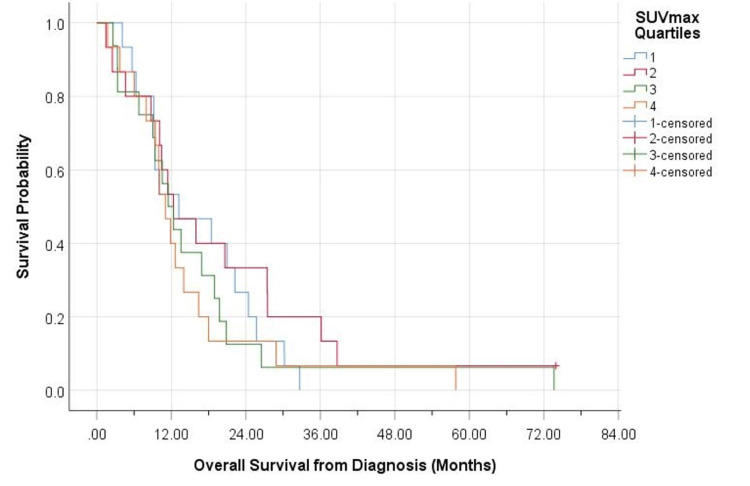
Relationship between SUVmax and overall survival from the date of diagnosis SUVmax, maximum standard uptake value

There was an inverse relationship between SUVmax and survival. When separating patients into quartiles based on their SUVmax, those classified into the lowest quartile (SUVmax ≤6.5) had the best overall survival when compared with those of higher quartiles (SUVmax 6.5 to 8.6, SUVmax 8.7 to 11, and SUVmax >11) (Figures 1, 2).

Multivariable analyses

We performed multivariable analyses taking into account age, sex, race, distant metastasis, tumor location, SUVmax, total bilirubin, CA 19-9, albumin, surgery, chemoradiation, and chemotherapy. In the analysis, SUVmax emerged as one of the independent predictors of survival (Table 2).

**Table 2:** Multivariable analyses of survival predictors PET/CT, positron emission tomography/ computerized tomography; SUVmax, maximum standard uptake value; CA 19-9, cancer antigen 19-9 level

Independent prognostic factors	Hazard ratio for survival calculated from PET/CT scan date (95% CI)	p-value	Hazard ratio for survival calculated from diagnosis date (95% CI)	p-value
Race:		0.007		0.003
-White	0.25 (0.09-0.68)		0.21 (0.07-0.58)	
-Other races	Reference		Reference	
SUVmax:		0.032		0.026
-≥8.7	2.26 (1.07-4.75)		2.36 (1.11-5.03)	
-<8.7	Reference		Reference	
Albumin level, per 1 g/dL increase	0.33 (0.14-0.80)	0.015	0.38 (0.16-0.92)	0.033
Age, per 1 year increase	1.56 (1.09-2.22)	0.014	1.61 (1.13-2.28)	0.008
CA 19-9 level, per 10,000 increase	1.69 (1.30-2.22)	<0.001	1.71 (1.31-2.23)	<0.001

Based on survival calculated from the date of the PET/CT scan, a higher SUVmax significantly increased the risk of death, with hazard ratio (HR) 2.26, p=0.03. Based on survival as calculated from the date of diagnosis, higher SUVmax also significantly increased the risk of death, with HR 2.36, p=0.03. Furthermore, the multivariable analyses showed that White race, higher albumin level, younger age, and lower level of tumor marker CA 19-9 were significantly predictive of better survival.

## Discussion

In this single-institution, retrospective cohort study, we found that the highest SUVmax obtained from PET/CT scan conducted near the time of systemic therapy initiation held a significant prognostic value for patients with advanced cholangiocarcinoma. Patients with higher SUVmax had poorer survival than their counterparts. The prognostic value was observed regardless of whether survival was calculated from the date of the first PET/CT scan or from the date of diagnosis.

To our knowledge, this is the first study quantifying the prognostic value of PET/CT scans among patients with exclusively advanced cholangiocarcinoma conducted in a Western country. We performed a literature search on MEDLINE (PubMed®) for studies of PET/CT scan in cholangiocarcinoma published from 2013 to 2022. Including the present study, there were 10 relevant publications. The findings from our study appear to be in line with previous studies conducted in Asian countries or in earlier-stage disease in that PET/CT carries a significant prognostic value, albeit at varying SUVmax cut-off levels, ranging from 5.0-8.0 (Table 3).

**Table 3:** Summary of publications on PET/CT in early-stage or advanced cholangiocarcinoma *early SUVmax obtained at 60 minutes after FDG injection; ^†^TLR, tumor liver ratio, was calculated from SUVmax divided by SUVmean of normal liver PET/CT, positron emission tomography/ computerized tomography; SUVmax, maximum standard uptake value

First author, year [reference]	Diseases	Country	Total patient number	Patients with advanced cholangiocarcinoma	Prognostic predictor
Current study, 2022	Cholangiocarcinoma	USA	61	61	SUVmax ≥8.7
Yoh et al., 2019 [12]	Cholangiocarcinoma	Japan	82	0	SUVmax ≥8.0
Kim et al., 2020 [13]	Cholangiocarcinoma	Korea	234	114	SUVmax ≥5.0
Hwang et al., 2021 [14]	Cholangiocarcinoma	Korea	55	41	SUVmax* ≥7.2
Sabaté-Llobera et al., 2019 [15]	Cholangiocarcinoma	Spain	60	33	SUVmax ≥6.6
Lim et al., 2019 [16]	Hepatocellular-cholangiocarcinoma	Korea	46	11	TLR† >3.4
Cho et al., 2015 [17]	Cholangiocarcioma, gallbladder, ampulla	Korea	106	Not reported	SUVmax ≥7.5
Lee et al., 2017 [10]	Cholangiocarcinoma	Korea	76	60	SUVmax ≥7.3
Park et al., 2014 [7]	Cholangiocarcinoma, gallbladder, ampulla	Korea	64	0	SUVmax >5.0
Lee et al., 2013 [18]	Cholangiocarcinoma, gallbladder	Korea	61	Not reported	SUVmax ≥5.5

Most of the previous studies were conducted in Asian countries where cholangiocarcinoma is an endemic disease, with the exception of one study, which was conducted in Spain. The SUVmax values used for prognostic analyses in these studies may differ due to heterogeneity in patient characteristics or imaging techniques [19]. Previous studies have shown that tumors with higher SUVmax are associated with distant metastasis and increased tumor burden, therefore leading to poorer prognosis [20].

Beyond SUVmax, our study has identified other prognostic factors. These were age, albumin, CA19-9, and race. Age is an unsurprising factor given the constitutional changes of aging and general trends of morbidity and mortality. Previous studies have also shown serum albumin level, which may indicate nutritional status, and serum CA 19-9, which may indicate tumor burden, to be prognostic for cholangiocarcinoma [21-23]. CA 19-9 was predictive of survival after resection of extrahepatic cholangiocarcinoma, ranging in cut-off value from 30 to 300 U/mL. Interestingly, in our study, patients with their race identified as White had a more favorable survival. This observation is in line with a previous study from the SEER database in which White patients had better survival than Blacks or Hispanics [24]. Race represents a constellation of socioeconomic and genetic influences. It is possible that White race may be associated with less aggressive disease. However, it is also possible that a differential treatment pattern is at play. White patients are significantly more likely to receive guideline-recommended treatment than non-White patients [25]. Given that up to 35% of cholangiocarcinoma in the United States occurs among ethnic minorities [26], future research will be necessary to understand the underlying mechanism between race and survival in this disease.

This study is limited by a number of factors. As a retrospective study, our results may be subjected to documentation deficiency or missing data, thus reducing the number of patients with analyzable data, although this issue is somewhat mitigated by the availability of electronic medical records and the study period, which is restricted to after 2009. Furthermore, it should be noted that we did not perform an analysis to identify an optimal cut-off value of SUVmax for survival prediction. For this, a larger study will be necessary to achieve adequate statistical power to define an optimal cut-off value. Given the rarity of cholangiocarcinoma in our patient population, we are limited by sample size. Nevertheless, in our study, events or deaths have occurred in all but one patient, thus substantially improving the statistical power.

## Conclusions

Our study, which was conducted among a predominantly Caucasian patient population, characterized the inverse association between SUVmax measured before systemic therapy and survival outcomes among patients with advanced cholangiocarcinoma in a similar fashion as previous studies conducted mostly in Asian countries. As baseline SUVmax increased, the overall survival, either calculated from the date of diagnosis or PET/CT scan progressively deteriorated. Based on our analyses, we concluded that PET/CT scan among patients with advanced cholangiocarcinoma can be useful clinically for prognostic purpose. This findings, however, should be interpreted in the context of limited efficacy of available systemic therapy during the study period. Future studies will be needed to identify an optimal cut-off value for further refinement in prognostication in this patient population.
